# Dentigerous Cysts

**Published:** 1871-12

**Authors:** W. P. Bowles


### Selections.

### DENTIGEROUS CYSTS.

BY W. P. BOWLES, M. D.

**An Essay to which was awarded the first prize of the Boylston Medical Society for 1871.**

This is not an affection which demands our time by its frequency or fatality. Yet its occurrence is sufficiently common to give its study practical interest, and, when present, it requires an intelligent interference to accomplish a cure. Coming, as this tumor does, in the most conspicuous part of the body, it not only encroaches upon the two great cavities of the face and impairs their functions, but thrusts out the lips or cheek, where concealment is impossible, to form an ugly and alarming deformity; and this, too, usually during a period when personal beauty is valued most highly. The surgeon who is familiar with this disease and can eliminate the various growths which simulate it, can promise to his patient not only life and comfort, but a restoration of comeliness. The chief interest of the disease, however, is not in the practical view just mentioned, but involves considerations which include also the structure and development of the organs affected. Dentigerous cysts owe their possibility to these elements, and are therefore peculiar to the jaws; they are extraordinary developments of cavities which, at a certain period of fœtal life, exist normally, and cover the teeth as they lie imbedded in the substance of the maxillary arches; an abnormal action of the secreting power which these cysts possess, in fact causes the disease which we are considering. The cysts are not at all related in their nature to those cavities of other bones with which, until recently, they have been confounded, nor even with certain other cystic troubles of the jaws themselves, from which even now they are scarcely separated. The development of the parts concerned must, therefore, be studied before the disease itself.

Very early in fœtal life, before the human embryo attains an inch in length, even while the nose and mouth are still a common chasm, a thickening of the membrane of the maxillary arches forms a semi-circular ridge around their corresponding margins. The epithelium covering this ridge now proliferates, and along the summit of the ridge *several* layers of cells, instead of *one*, are found which indent the underlying substance, dividing the ridge into two ("dental ridges") by a shallow groove ("dental groove"). From the bottom of this groove a narrow epithelial fold grows deeply into the tissues beneath it, and is called the "enamel germ." These changes, according to the more recent observers, appear in the deeper layers of the epithelium only, while the free surface is kept nearly in the same place by the accumulation of its cells. The enamel germ next widens at the bottom, and becomes divided into segments corresponding with the number of the temporary teeth. Each of these divisions, which are called "enamel organs," is somewhat flask-shaped and connected with the groove above by a narrow neck, the relic now of the enamel germ. A papilla, called the "dental germ," soon grows into the enamel organ from below, carrying, as it advances, the floor of the latter upon its summit; clothed with this it projects boldly into the "flask," invaginating its lower half, and there assumes the likeness of a tooth, with the enamel organ, now something of a "meniscus" in shape, resting upon its crown. The narrow isthmus reaching to the surface of the jaw then contracts and disappears, a vascular and fibrous envelop* surrounds the parts described, and is itself encompassed by the ossifying jaw. Thus the tooth is isolated from the mouth and from its fellows, and enclosed in solid bone in most close connection with an equally isolated epithelial sac.†

Those cells of the dental germ which lie immediately

* "Dental sac." Waldeyer, however, describes this as "richer in cells and vessels," and says that it disappears early.—Stricker's Histology, vol. I., p. 488 (Syd. Soc. Trans.

† The object of nature in thus enclosing the dental organs seems to be to accomplish the formation of the enamel, for in certain fishes whose development does not extend beyond the "follicular" stage of Goodsir, dentine only, and no enamel, is found upon the pulp.—Owen, Odontography p. 17.

beneath its surface are next developed into a well-marked layer of bone-forming elements,* by means of which the dentine is produced, and soon forms a thin shell over the crown of the papilla, and increases in thickness, growing toward the centre of the tooth by successive layers of the same "odontoblasts."

Meanwhile, very great changes have taken place in the enamel organ. Its upper wall, but slightly altered, still consists of short and cubical cells. Its interior presents a web of stellate cells, converted from the accumulated epithelium, and widely separated in "a rich albuminous fluid." The layer enveloping the dentine would not now be recognized; its cells have become prismatic, regular and very long, their nuclei have advanced toward their superficial extremities (i. e., next the enamel pulp), their ends appear to be open. It is, however, continuous with the outer wall at its border, find the true epithelial covering of the remarkable papilla beneath it.† An intermediate layer‡ of loose and rounded cells lies between this and the stellate forms in the middle. The enamel begins on the surface of the dentine by the calcification of these prismatic cells, and increases outward (in an opposite direction from the progress of the other), either by their elongation or successive replacement by the rounded ones next to them. Thus the growing enamel encroaches upon the enamel organ and is nourished by it.§ The latter wastes as the other increases,

---

* "Odontoblasts," Waldeyer. "Dentinal layers," Owen.

† Although it would be inferred from the older writers, and especially from every picture which I have seen, that the enamel membrane is not connected with the dental germ, yet it seems to me to be its own true epithelium, and that its free surface, properly speaking, is that which faces the enamel pulp; also, that this latter is the only "cavity" in the dental sac. The interspace always figured between this membrane and the dentine is probably diagrammatic. If this be so, we have here the anomalous condition of a superficial membrane cut off from supplies below (by the hardening of the underlying dentine) and nourished from its homologically free surface, that facing the enamel pulp.

‡ "stratum intermedium."

§ This is the view expressed by most authors; but Waldeyer says the office of the enamel pulp is only "transitory and mechanical;" to fill the cavity reserved for the tooth.—Stricker, op. cit. p. 485.

The "membrana præformativa," whose function, and even presence or absence, were for a time the cause of so much discussion and difference of opinion as even nearly to deprive their enamel cells of their and the tooth advances toward the gum, until, finally, the the pulpy part disappearing, its upper wall lies upon the surface of the enamel and hardens on it as the "cuticula dentis."—Stricker, op. cit. p. 485.

The cement is formed below the margin of the enamel by the ossification of the dental capsule. ("From the connective tissue of the dental alveoli."—Waldeyer.)

In the course of time the fang develops and elongates, the jaw and gum dissolve before the advancing tooth, and this at last appears in the cavity of the mouth.

From the inner side of the neck of each of the enamel organs which serve the temporary teeth, a second pouch is given off, which afterward serves the corresponding permanent tooth in a similar course of development. There always remains, however, an unossified cord extending from this capsule, either to the gum or to the socket of the temporary tooth from which it sprung; thought to be a relic of the neck of the enamel organ*—a sort of umbilicus.

The first permanent molar is formed independently from the milk set, the second from this by the budding process just described for the permanent teeth generally, and the third in like manner from the second. These in this respect resemble three generations of teeth, which, however, do not succeed each other, because the growth of the jaw at this place allows them all to coexist †

The growth and eruption of the permanent teeth are governed by the same rules as those of the temporary teeth; the process, however, is embarrassed and rendered difficult by the presence of the latter. The temporary alveoli are invaded by the new teeth; the temporary roots dissolve before them, and, at length, only a simple crown falls away and a new one has taken its place.

The jaws, therefore, during childhood, especially in its

---

traditional office, is, I think, best explained by Waldeyer as the "youngest softish layer of the enamel," and not a separate membrane.—Stricker, vol. i. p 487.

* "Either because it is to become useful as a 'gubernaculum,' and its bony canal as an 'iter dentis,' or more probably in virtue of the general law that parts or organs which once acted an important part, however atrophied they may afterward become, never wholly disappear, while they do not interfere with other parts or functions."—Goodsir, Edin. Med Jour., 1838, p 28.

† But where they are unable to do so, the posterior may cause absorption and loosening of the anterior, as if it were merely its temporary predecessor.

early part, are full of these remarkable organs in active process of growth, and crowded together in such apparent confusion that the wonder is that accidents to their development do not oftener occur—that the teeth are as regular as they are.

The literature of dentigerous cysts is meagre and unsatisfactory. Many of the older writers have failed to mention them at all, while the rest have hopelessly mixed their cases in a confused mass of "spina ventosa" and "osteo-sarcoma." Dupuytren mentions one undoubted case,* and several others which may have been such, but no reference is made to teeth in connection with them, except that one "was caused by the incomplete extraction of a canine."—P. 136. His directions for treatment, however, are excellent; better, in fact, than those of some more recent authors, many of whom have added nothing to his account.

Stanley quotes two cases,† besides others similar to those of Dupuytren; he follows him in pathology and treatment, but recognizes more fully than he the distinction between cysts in the jaws and the various fluid cavities of other bones, yet fails to see the difference between the true dentigerous cyst and the one "caused by the irritation of a carious or misplaced tooth, and commencing near the extremity of the fang."—P. 300.

Warren gives some interesting cases (none, however, proved to be dentigerous), which he treated admirably and successfully, part of which he considered to be due to trouble at the roots of certain teeth.‡ March relates cases similar to Warren's.§ Jourdain (as quoted by Salter)‖ had three genuine cases. Other observers have each reported an instance, as may be seen in the Appendix. Salter, in Holmes' Surgery,¶ has given the best account of the disease which the writer has seen, and has collected quite a number of cases for illustration, besides giving two from his own practice. More recently, Heath,** in his work on the Jaws, devotes several pages to it, and gives three or four excellent

‖ Holmes' Surgery, vol. iv. ¶ Ibid.

** Heath on Injuries and Diseases of the Jaws.

drawings of specimens, but adds nothing new in theory or treatment.

The accounts in the common text-books are only compilations, and all inferior to those of the authors named. Glaswald is said to have written a very fine thesis upon it. It is not a little surprising so many errors should have occurred in the well written article* of Salter, in which he describes two additional cases.

*Clinical History.*†—This is, perhaps, the most neglected part in the recorded cases; many entirely fail to give the history of the case. Where it is given, it seems to be simple and easily told; a slow, nearly painless swelling of a portion of the jaw appears and increases, and a certain irregularity (to be referred to again) is noticed in dentition. What distress there is—usually slight, often none—is due to the tension and mechanical inconvenience of the new growth. In most cases this is all. The growth may be uniform and steady, or its progress may be retarded and again make fresh advances. It never diminishes. It may open in the mouth or through the cheek, causing either a permanent fistula, or it may close and open alternately. The wall of the antrum is usually compressed, rather than broken through; but this accident undoubtedly happens, and it often is impossible at the time of operation to say whether the cavity opened is the antrum or separate from it. Inflammation and suppuration often occur, but are not peculiar to dentigerous cysts. Such cases are apt to be painful. The size of the cavity varies, from that of an orange down.

The liability to cancerous degeneration is not so much feared now as formerly,‡ but that a fibrous growth occasionally follows has been observed in certain cases of cysts; it is doubtful, however, if such cases were actually dentigerous cysts.§

---

* Guy's Hosp. Rep., 3d Ser., vol. xv, 1870.

† While the opinions of authors have been freely used in the following paragraph, more weight has been given to the teachings of the cases themselves. On this account some of the following statements will be found different from the usual accounts, but a reference to the table will sustain them.

‡ "Le marche des kystes ossieux est generalement lente. Au bout d'un temps plus ou moins long ils passent a le degenerescence cancereuse, surtout ceux dont les produits sont fibro-celluleux.—Dupuytren, Leçons, etc., t. ii. p. 140.

§ The prognosis, says Mr. Syme, is more favorable if a tooth be found in the cyst; it is then almost certain that it will contract and heal.

Case No. 29, which occurred at the Boston City Hospital, in Dr. Thorndike's service, while the writer was his assistant, may be read as a typical example of the simple form of the disease. This, with Nos. 27 and 28, which occurred in his private practice, and No. 26, from that of Dr. Cheever, have not been published before, and are now presented with the permission of these gentlemen.

There are certain cases accompanied by a considerable amount of pain, either from the very first, or coming on after a little, and lasting until interfered with. These either arise from a blow or injury, in cases where the errant tooth is pushing away obstructions to its progress, or such as are accompanied by some less usual complication.

The diagnosis requires, first, the recognition of a hollow growth; and, secondly, the knowledge that it contains a tooth. The first is accomplished by physical examination, and would seem easy enough; yet a large proportion of the cases of removal of the bones for this disease were done through a mistake on this very point. The history of the case is not essential to this examination; yet an account of much pain, or cancerous tendency, would, of course, receive attention. An irregularity of its surface, polypi of the nose or pharynx, or a fungous appearance, are indicative of a solid tumor. A painless swelling of either jaw, of not very rapid growth, should always suggest the possibility of a cyst, and lead to a careful examination of every part of its surface, both over *the cheek and within the mouth.** Fluctuation will usually be detected in certain places at least, although the greater portion may be bony and unyielding.† If these thinner spots be indented, perhaps they will return to their places with a crumpling sound, which is pathognomonic of a cystic growth; if any doubt remain, an ex-

---

This may occur in other cases, but it has been noticed that cysts, after being opened, are not unfrequently followed by the formation of a solid tumor. I could mention to you cases where I have opened cysts containing nothing but serum, in the place of which solid tumors have afterward been formed.—Lancet, March 10, 1855, p. 253.

* "They are generally at some parts as hard and unyielding as the bone, so that if the explanation be limited to these the mass will be supposed to be solid."—Lyme, op. cit, p. 253.

† Judging from the *non-ossification of the old "gubernaculum"* and the cases which I have seen, I shall venture to say that that portion of the wall under the gum where the *missing tooth should normally have appeared* will always be found *membranous or else very thin.*

ploratory puncture, which is perfectly harmless, should always be made, as Dupuytren has well insisted: "Je regarde une crepitation legere comme un symptome pathoguomonique; ce signe merite beaucoup d'attention. S'il y a quelque doute on fait un ponction exploratrice; cette ponction et la crepitation, sont deux symptomes qui ne laissent aucun doute sur l'existence des kystes de cette nature."

The elimination of fluid accumulations in the antrum, old alveolar abscess or cysts containing blood, serum, or other matter, but not teeth, is more difficult. A decision from the above evidence alone would be impossible. There is one diagnostic mark, however, for these cases, which is of the highest value when it can be proved: this is *the absence of a permanent tooth* (which has never been removed) *some time after its appearance is due, in the neighborhood of the cyst, and whose place is quite likely to be filled by its temporary predecessor.* This condition in the mouth, in connection with the swelling, would be an almost positive indication of its nature; while, on the other hand, if all the teeth are present and normal, it is one of the other diseases.*

The predisposing and the exciting causes of this affection should be separately studied. The presence of a tooth in the jaw which has never erupted is, in fact, the only one cause of the disease, since youth and other conditions which have been considered as such are only accidental. The teeth concerned are of the permanent set† and usually delayed in the jaw, either in consequence of the irregular or non development of their fangs, their deep situation, or wrong direction, or else of the obstinate persistence of the temporary teeth in advance of them.

The exciting causes are often unknown, and probably various. The attempts of the impacted teeth to reach the surface have an exciting action in certain cases; but on the other hand, such teeth may lie quiet in the jaw during the entire life, and cause no trouble, or may be found enclosed in a cyst after all attempts at growth had been given up for

* Rare exceptions may occur to both these statements. Teeth may be indefinitely impacted or undeveloped, and yet produce no disturbance or may be absent altogether—the missing member never having had an existence—or they may be the innocent participants of an independent growth, as where they become involved in solid tumors. A supernumerary and a temporary tooth have each been found at fault.—(Appendix, Nos. 16 and 20.

† One exception, No. 16.

years. That the disease should follow the extraction or aching of carious teeth, is not remarkable, considering how common these are at all ages; yet such irritation in some cases may have been the exciting cause as well as the obstinate resistance of a milk tooth to the advance of its follower. In the first and second cases given in the tables the disease was evidently excited by the blows which preceded. The additional presence of undeveloped teeth in the jaw is, of course, essential in all these cases, for without these the affection *can not exist.—Medical and Surgical Journal.*

(To be continued.)

